# Water-assisted oxidative redispersion of Cu particles through formation of Cu hydroxide at room temperature

**DOI:** 10.1038/s41467-024-47397-z

**Published:** 2024-04-08

**Authors:** Yamei Fan, Rongtan Li, Beibei Wang, Xiaohui Feng, Xiangze Du, Chengxiang Liu, Fei Wang, Conghui Liu, Cui Dong, Yanxiao Ning, Rentao Mu, Qiang Fu

**Affiliations:** 1 Department of Chemical Physics, University of Science and Technology of China, Hefei, China; 2 State Key Laboratory of Catalysis, Chinese Academy of Sciences, Dalian Institute of Chemical Physics, Dalian, China; 3 Center for Transformative Science, ShanghaiTech University, Shanghai, China; 4 Faculty of Environmental Science and Engineering, Kunming University of Science and Technology, Kunming, China

**Keywords:** Heterogeneous catalysis, Nanoparticles, Materials for energy and catalysis

## Abstract

Sintering of active metal species often happens during catalytic reactions, which requires redispersion in a reactive atmosphere at elevated temperatures to recover the activity. Herein, we report a simple method to redisperse sintered Cu catalysts via O_2_-H_2_O treatment at room temperature. In-situ spectroscopic characterizations reveal that H_2_O induces the formation of hydroxylated Cu species in humid O_2_, pushing surface diffusion of Cu atoms at room temperature. Further, surface OH groups formed on most hydroxylable support surfaces such as γ-Al_2_O_3_, SiO_2_, and CeO_2_ in the humid atmosphere help to pull the mobile Cu species and enhance Cu redispersion. Both pushing and pulling effects of gaseous H_2_O promote the structural transformation of Cu aggregates into highly dispersed Cu species at room temperature, which exhibit enhanced activity in reverse water gas shift and preferential oxidation of carbon monoxide reactions. These findings highlight the important role of H_2_O in the dynamic structure evolution of supported metal nanocatalysts and lay the foundation for the regeneration of sintered catalysts under mild conditions.

## Abstract (web summary)

Redispersion of sintered metal species requires high temperatures in reactive atmospheres. Here, the authors report room temperature redispersion of supported Cu particles via formation of mobile hydroxylated Cu species induced by gaseous H_2_O and anchoring of the Cu species by surface OH groups.

## Introduction

Supported metal nanocatalysts are commonly employed in heterogeneous catalysis, while sintering of supported metal species often happens during high-temperature reactions leading to catalyst deactivation^1–5^. To address this issue, various redispersion strategies have been developed with the aim of reversing the sintering and revitalizing the active metal species^6–11^. The redispersion of metal species typically involves detachment of metal atoms from larger particles, followed by surface or vapor-phase migration and final capture by surface anchoring sites^10,12^. The success of redispersion largely depends on metal-support interaction, determining thermodynamic favorability of the process^8–10,13–15^. Therefore, supports with various surface defects or surface functional groups (oxygen vacancy, OH, heteroatom, etc.) are essential for anchoring mobile metal species through strong interaction between metal and support^8,9,13,14^. Furthermore, kinetic factors also play a pivotal role which cannot be underestimated. Thus, high-temperature treatments in specific gaseous environments (CH_3_I, O_2_, NH_3_, etc.) are often employed to enhance the mobility of metallic species across the support surface or through gas-phase migration^7,16–18^. Apparently, these redispersion processes require a considerable energy input. The quest for eco-friendly and energy-saving redispersion strategies remains an urgent priority^10^.

Owing to the low melting temperature of copper metal (1083 °C) and its low Hüttig and Tammann temperatures (174 and 405 °C, respectively), Cu-based catalysts are susceptible to sintering, thereby limiting their industrial applications^19,20^. However, the high mobility of Cu atoms also allows the facile redispersion of Cu particles under relatively mild conditions^21,22^. In recent years, the structural change of Cu nanocatalysts at room temperature (RT) has been occasionally reported^23–28^. The incorporation of Cu atoms from Cu nanoparticles (NPs) into silica matrix^24^, intercalation of Cu atoms from bulk Cu into layered transition metal dichalcogenides^25^, and disintegration of Cu nanoparticles into single atoms on N-doped carbon supports^23,27^ have been observed under ambient conditions, which are driven by coordination of Cu atoms with surface atoms of the support e.g., O, Si, S, and N. Sun et al. find that pre-adsorbed H_2_O on ZnO surface can also promote redispersion of Cu particle into single atoms and few-atom Cu clusters at RT. Despite these interesting findings the mechanism underlying the redispersion process and the dynamic interaction between gaseous atmosphere and metal atoms/support during the processes remain unclear, thus necessitating in-depth studies.

In this work, the dynamic behavior of supported Cu NPs in various atmospheres at RT has been investigated by a variety of characterization techniques including high-angle annular dark-field scanning transmission electron microscopy (HAADF-STEM), X-ray photoelectron spectroscopy (XPS), X-ray absorption spectroscopy (XAS), and ultraviolet-visible (UV-Vis) diffuse reflectance spectroscopy. The results reveal the spontaneous redispersion of aggregated Cu particles into Cu single atoms and ultrasmall clusters on γ-Al_2_O_3_ in humid air and at RT. H_2_O is found to promote the formation of mobile hydroxylated Cu species in O_2_, pushing the diffusion of Cu atoms. Meanwhile, the enriched surface OH groups of the support in a humid atmosphere provide anchoring sites to pull the diffusing hydroxylated Cu atoms. Redispersion of Cu particles into Cu single atoms and clusters under mild ambient conditions are achieved by synergizing the thermodynamic and kinetic effects of H_2_O. The generated Cu single atoms and clusters on γ-Al_2_O_3_ and CeO_2_ supports exhibit high activity in reverse water gas shift (RWGS) and preferential oxidation of carbon monoxide (CO-PROX) reactions, respectively. Moreover, the deactivated Cu catalysts after the reactions can be facilely reactivated by exposing to O_2_-H_2_O at RT.

## Results

### Spontaneous redispersion of Cu NPs in air at RT

**Fig. 1:**
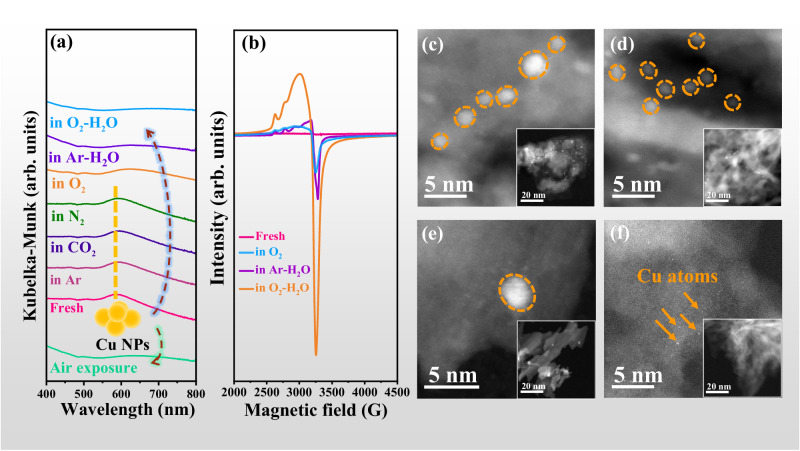
Spontaneous redispersion of Cu NPs at RT. **a** In-situ UV-Vis spectra of 2Cu/AlOOH-900 treated in different atmospheres (Air, Ar, CO_2_, N_2_, Ar-H_2_O, O_2_, and O_2_-H_2_O) for 30 min. **b** EPR spectra of 2Cu/AlOOH-900 before and after treatment in O_2_, Ar-H_2_O and O_2_-H_2_O atmospheres for 24 h. HAADF-STEM images with high-magnification and low-magnification (insets) of (**c**) 2Cu/AlOOH-900, 2Cu/AlOOH-900 treated in (**d**) O_2_, (**e**) Ar-H_2_O and (**f**) O_2_-H_2_O for 24 h.

γ-Al_2_O_3_ supports were prepared by calcination of pseudo-boehmite at different temperatures as confirmed by X-ray diffraction (XRD) in Fig. S1, which are denoted as AlOOH-*T* (*T* represents calcination temperature, *T* = 500 and 900 °C)^14^. Cu NPs supported on γ-Al_2_O_3_ (2 wt.%) were prepared by wet impregnation method and denoted as 2Cu/AlOOH-*T* (details seen in Methods). The absorption band around 570 nm in UV-Vis spectra (Fig. 1a) and diffraction peaks at 43.3 and 50.4° in XRD patterns (Fig. S2) are characteristic for metallic Cu NPs^29–31^. Interestingly, these signals for Cu NPs disappear after exposure to air for one week (Fig. 1a and Fig. S2). A new broad absorption peak around 600–800 nm assigned to Cu^2+^ species^32^ appears and no diffraction peaks of Cu species are present, indicating that Cu NPs may transform into smaller Cu species in air.

In-situ UV-Vis experiments were conducted to monitor the structural evolution of Cu species in 2Cu/AlOOH-900 when exposed to each component of air at RT including N_2_, Ar, CO_2_, O_2_, Ar-H_2_O and O_2_-H_2_O. The fresh sample was used for each in-situ characterization. Figure 1a shows that the characteristic adsorption peak of metallic Cu NPs remains unchanged in N_2_, Ar, and CO_2_, yet is markedly diminished in O_2_, Ar-H_2_O and O_2_-H_2_O gases. The observation suggests that the redispersion of Cu NPs in air may be caused by the oxidizing gas components including O_2_, H_2_O, and both together.

It is known that atomically dispersed Cu^2+^ species with an unpaired electron in the *d*_*x*_^2^_−*y*_^2^ orbital are active for electron paramagnetic resonance (EPR) while Cu^2+^ ions in crystalline CuO are EPR-inactive due to the strong antiferromagnetic coupling^33–36^. No obvious EPR signal is observed for 2Cu/AlOOH-900 (Fig. 1b), indicating the absence of highly dispersed Cu^2+^ species in the fresh sample. In contrast, much strong EPR signals around 3300 G are detected in 2Cu/AlOOH-900 after treatment in O_2_-H_2_O for 24 h at RT as well as in Ar-H_2_O and O_2_ (Fig. 1b), implying that O_2_-H_2_O treatment generates highly dispersed Cu^2+^ species^35^.

HAADF-STEM was then used to determine the size of Cu species treated in different atmospheres. Cu NPs with an average diameter of about 3 nm are observed in the fresh 2Cu/AlOOH-900 sample (Fig. 1c and corresponding inset). After treatment in O_2_ and Ar-H_2_O at RT for 24 h, Cu clusters with size of 2 nm are still observed (Fig. 1d, e and corresponding insets). In contrast, only Cu single atoms are imaged in the O_2_-H_2_O treated sample (Fig. 1f and corresponding inset). These results illustrate that Cu NPs are completely redispersed into Cu single atoms in an O_2_-H_2_O atmosphere but only partly redispersed in O_2_ or Ar-H_2_O at RT for 24 h.

### Effect of H_2_O on Cu redispersion process

**Fig. 2:**
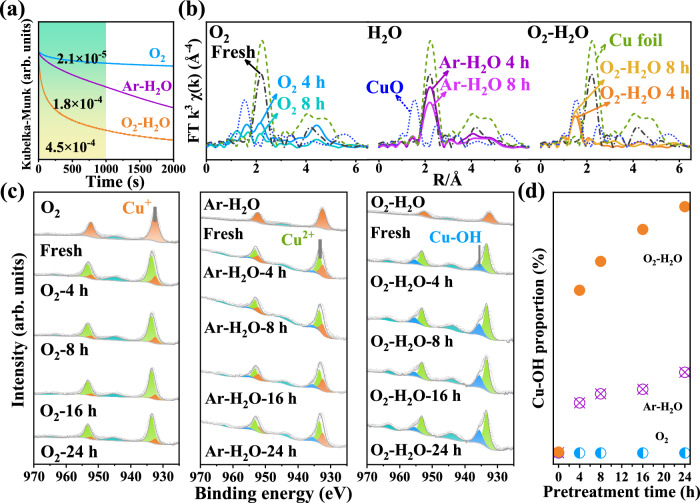
H_2_O effect on redispersion of Cu NPs at RT. **a** In situ UV-Vis spectra of 2Cu/AlOOH-900 treated in different atmospheres. **b** Quasi in-situ Fourier-transforms of k^3^-weighted Cu K-edge EXAFS spectra of 2Cu/AlOOH-900 treated in O_2_, Ar-H_2_O and O_2_-H_2_O for 4 h and 8 h, as well as standard samples of Cu foil and CuO. **c** Quasi in-situ Cu 2*p* XPS spectra of 2Cu/AlOOH-900 treated in O_2_, Ar-H_2_O and O_2_-H_2_O for 4, 8, 16 and 24 h. **d** Changes in the proportion of Cu-OH species over time in different atmospheres calculated based on Cu 2*p* XPS spectra.

Time-dependent in-situ UV-Vis spectroscopy was employed to monitor the evolution of Cu species in O_2_, Ar-H_2_O and O_2_-H_2_O atmospheres. The redispersion rate of metal NPs can be represented by the slope of the kinetic curve (-ΔKM/Δ*t*)^14,37^. As shown in Fig. 2a, the redispersion rate at early stage (1000 s) in various atmospheres follows the sequence of O_2_-H_2_O (4.5 × 10^−4^) > Ar-H_2_O (1.8 × 10^−4^) > O_2_ (2.1 × 10^−5^), indicating that O_2_-H_2_O atmosphere accelerates the redispersion process. Subsequently, quasi in-situ XAS experiments were conducted to identify the chemical state of Cu in O_2_, Ar-H_2_O and O_2_-H_2_O atmospheres. As shown in Fig. 2b and Fig. S3, the main peak centered at 2.2 Å in extended X-ray absorption fine structure (EXAFS) spectra of 2Cu/AlOOH-900 is assigned to Cu-Cu bond similar to that in Cu foil, implying that metallic Cu dominates in the fresh sample, which agrees with the Cu K-edge X-ray absorption near-edge structure (XANES) results (Fig. 2b, Figs. S3 and S4)^38,39^. The peak of Cu-Cu bond disappears in O_2_-H_2_O for 4 h while only gets weaker in O_2_ and Ar-H_2_O even if the treatment time is extended to 8 h (best-fit parameters summarized in Table S1). Meanwhile, a peak around 1.5 Å appears (Fig. 2b and Fig. S3) which is the typical scattering feature of Cu-O coordination, accompanied by the obvious peak of Cu^2+^ species around 8996 eV in the XANES spectra (Fig. S4)^38,39^. The results further indicate that the redispersion of Cu NPs into Cu single atoms occurs more rapidly in O_2_-H_2_O, in accordance with HAADF-STEM and UV-Vis results.

It has been revealed that surface hydroxyl (OH) groups significantly affect the redispersion of metal NPs^14,40^. According to the exchange reaction D_2_ + OH → OD + HD, the HD signal in the H-D exchange experiment can be used to characterize OH content on support surface^41^. As shown in Fig. S5, H-D exchange results demonstrate that there is no significant difference in surface OH content of γ-Al_2_O_3_ support treated in Ar-H_2_O and O_2_-H_2_O atmospheres. Thus, support surface hydroxylation is not the decisive reason for the different redispersion behavior of Cu NPs under Ar-H_2_O and O_2_-H_2_O atmospheres.

Quasi in-situ XPS experiments were thus conducted to identify the surface Cu species after treatment in the various atmospheres. Cu 2*p*_3/2_ peak located at 932.4 eV is observed in the fresh 2Cu/AlOOH-900 sample (Fig. 2c), which is assigned to Cu^+^/Cu^0^ species^42,43^. The kinetic energy of the main Cu L_3_VV Auger peak at 916.6 eV and a weak peak around 922.0 eV indicate that Cu^+^ and a small amount of Cu^0^ species coexist on the surface of fresh 2Cu/AlOOH-900 sample^43^ (Fig. S6). After treatment in O_2_, Ar-H_2_O and O_2_-H_2_O atmospheres for 4 h, the main Cu 2*p*_3/2_ peak shifts to 933.4 eV, characteristic for Cu^2+^ species in CuO, which is further confirmed by Cu L_3_VV Auger peak at 918.1 eV^43^. While the peak at 932.4 eV indicates that Cu^+^ species still exist in the samples treated in O_2_ and Ar-H_2_O for 4 h, consistent with Cu L_3_VV Auger peak at 916.6 eV (Fig. S6)^43^. Interestingly, a Cu 2*p*_3/2_ peak at 935.6 eV and a Cu L_3_VV Auger peak at 914.4 eV appear in the samples treated in Ar-H_2_O and O_2_-H_2_O for 4 h, corresponding to hydroxylated Cu (Cu-OH) species (Fig. 2c)^43^. As the treatment time extends from 4 to 24 h, the proportion of Cu-OH in Ar-H_2_O and O_2_-H_2_O gradually increases from 4.4% to 7.1% and from 14.3% to 21.7%, respectively. The proportion of Cu-OH species related to total Cu species in the O_2_-H_2_O treated sample is much higher than those in the samples treated in Ar-H_2_O and O_2_, indicating that Cu-OH species are easily formed in O_2_-H_2_O (Fig. 2d). It is worth noting that if Cu NPs are firstly oxidized to CuO in O_2_ and then exposed to Ar-H_2_O at RT for 24 h, the proportion of Cu-OH species reaches 10.4%, which is much lower than that of Cu NPs exposed to O_2_-H_2_O (21.7%), but higher than Cu NPs directly exposed to Ar-H_2_O (7.1%) (Fig. S7).

The above results suggest that spontaneous redispersion of Cu NPs in O_2_-H_2_O may occur through the oxidation of Cu atoms into atomic Cu-O species, followed by quick hydroxylation into atomic Cu-OH species and final capture by the support. Furthermore, we find that the oxidative redispersion process relies on the size of Cu particles. The redispersion of micron-sized Cu particles is very slow compared with nano-sized Cu particles under the same conditions (Fig. S8). Overall, combining EPR, XAS and UV-Vis results, it can be reasonably inferred that the formation of Cu-OH species is the key to promoting the redispersion of Cu NPs.

### Effect of Cu precursors on the redispersion process

**Fig. 3:**
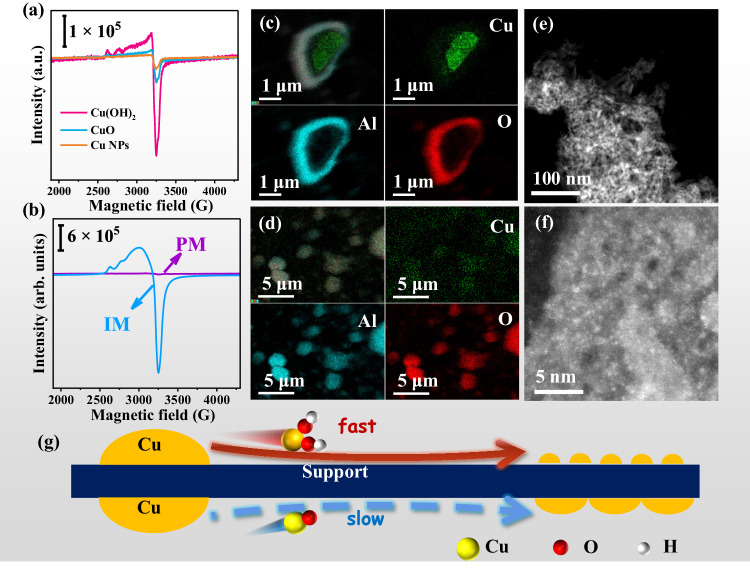
Effect of Cu precursors on redispersion at RT. **a** Quasi in-situ EPR spectra of physical mixtures of AlOOH-900 and different Cu precursors in Ar-H_2_O atmospheres for 24 h. **b** EPR spectra of Cu(OH)_2_-AlOOH-900 before (PM) and after water immersion (IM) for 24 h. EDX mapping images over Cu(OH)_2_-AlOOH-900 (**c**) before and (**d**) after water immersion for 24 h. **e, f** HADDF-STEM images of Cu(OH)_2_-AlOOH-900 after water immersion for 24 h. **g** Scheme of the effect of migration species on the redispersion process.

Commercial Cu, CuO, and copper hydroxide (Cu(OH)_2_) powders with similar particle size were mixed with AlOOH-900, and then treated in Ar-H_2_O at RT for 24 h to investigate the effect of Cu precursors on the redispersion process. As shown in Fig. 3a, a rather stronger EPR signal around 3300 G is detected using Cu(OH)_2_ as the precursor as compared to Cu and CuO, implying that Cu(OH)_2_ is easier to be redispersed. The above results confirm the important role of hydroxylated Cu-OH species in the Cu redispersion process.

Inspired by the crucial role of H_2_O in the redispersion process, we further investigated whether the redispersion process in gas-phase H_2_O could be extended to liquid-phase H_2_O. Cu(OH)_2_ powder was physically mixed with AlOOH-900 (denoted as Cu(OH)_2_-AlOOH-900) and immersed in liquid-phase H_2_O, followed by stirring for 24 h at RT. The obvious diffraction peak at 23.8° and the absence of EPR signal at 3300 G (Fig. S9 and Fig. 3b) in Cu(OH)_2_-AlOOH-900 sample indicate that only bulk Cu(OH)_2_ exists in the physical mixture^44^. Large Cu-based particles are also observed in scanning electron microscope and energy-dispersive X-ray spectroscopy (SEM-EDX) mapping images (Fig. 3c). After the water immersion treatment, the diffraction peak of Cu(OH)_2_ at 23.8° vanishes, and a strong EPR signal at 3300 G appears (Fig. S9 and Fig. 3b). The results indicate that Cu(OH)_2_ aggregates are redispersed into highly dispersed Cu species which are uniformly distributed on AlOOH-900 surface as shown by the SEM-EDX mapping images (Fig. 3d).

HAADF-STEM images (Fig. 3e, f) further confirm that clusters around 2 nm form after the water immersion. Significantly, EPR signal of the sample treated in liquid-phase H_2_O is much stronger than that in gas-phase H_2_O (Fig. 3a, b). The above results reveal that Cu-OH species rather than other Cu intermediates such as CuO_*x*_ dominate the fast redispersion process at RT (Fig. 3g), highlighting the indispensable role of H_2_O in the redispersion of Cu particles at RT. Apart from inducing the formation of Cu-OH species, H_2_O treatment would also cause the surface hydroxylation of the γ-Al_2_O_3_ support. H-D exchange results show about a two-fold increase in the OH content compared to O_2_ treatment (Fig. S10). Therefore, the impact of surface hydroxylation of the γ-Al_2_O_3_ support on metal redispersion may not be ignored and was investigated in detail.

### Role of surface OH groups in the Cu redispersion

**Fig. 4:**
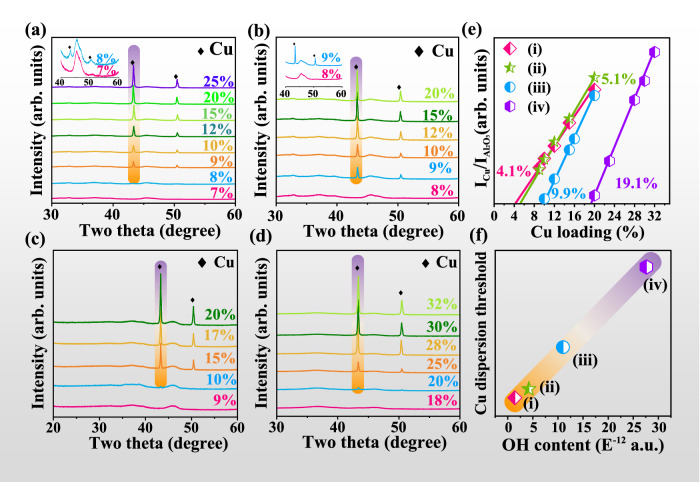
Effect of surface OH content of γ-Al_2_O_3_ on Cu dispersion threshold at RT for 24 h. XRD patterns of (**a**) *x*Cu/AlOOH-900 treated in O_2_ and partial magnification of XRD at 40–60° under 7 and 8 wt.% Cu loading (inset). **b** *x*Cu/AlOOH-900 treated in O_2_-H_2_O and partial magnification of XRD at 40–60° under 8 wt.% Cu loading (inset). **c** *x*Cu/AlOOH-900-H_2_O_2_ treated in O_2_ and (**d**) *x*Cu/AlOOH-500 treated in O_2_. **e** Relative intensity of Cu (111) diffraction peak vs. γ-Al_2_O_3_ (400) diffraction peak for various Cu/γ-Al_2_O_3_ samples derived from the XRD results. **f** Correlation between OH content and Cu dispersion threshold. **(i)** *x*Cu/AlOOH-900 treated in O_2_, **(ii)** *x*Cu/AlOOH-900 treated in O_2_-H_2_O, **(iii)** *x*Cu/AlOOH-900-H_2_O_2_ treated in O_2_ and (**iv**) *x*Cu/AlOOH-500 treated in O_2_.

For the spontaneous monolayer dispersion phenomenon proposed by Xie et al., active components will disperse onto support surface in the form of atoms or small clusters which cannot be detected by XRD^45^. The amount of dispersed active components as a monolayer is defined as the dispersion threshold, above which diffraction peaks of the active components can be observed in XRD^46–48^. The dispersion threshold of an adsorbate on a support can be determined by this well-established method^46^. For supported Cu samples treated in O_2_ at RT for 24 h (Fig. 4a), no diffraction peaks of Cu species are detectable when Cu loading is below 8 wt.%, confirming that all Cu species are well dispersed on γ-Al_2_O_3_. Above 8 wt.% Cu loading, additional small peaks at 43.3 and 50.4° are detected which are assigned to metallic Cu (PDF # 04-0836), and intensities of these peaks increase with the increased Cu loadings. Cu (111) peak intensity (*I*Cu) has been normalized by that of γ-Al_2_O_3_ (400) peak (*I*Al_2_O_3_) for each sample to obtain *I*Cu/*I*Al_2_O_3_. From the plot of *I*Cu/*I*Al_2_O_3_ with the Cu loading, the correlation line intersects the *x*-axis at a point which corresponds to the Cu dispersion threshold at 4.1 wt.% (Fig. 4a, e). The dispersion threshold of O_2_-H_2_O treated sample is found to be 5.1 wt.% using the same method (Fig. 4b, e). The results suggest that the addition of H_2_O has effectively increased the dispersion threshold of Cu species on γ-Al_2_O_3_ surface, which is proposed to result from the enriched surface OH groups as discussed below.

Al_2_O_3_ supports with different OH contents (Fig. S10) were used to explore the crucial role of surface OH groups in the spontaneous redispersion of Cu NPs. Surface OH content of γ-Al_2_O_3_ was tuned through treatment in H_2_O_2_ solution or calcination of the pseudo-boehmite precursor at various temperatures^14^. As the OH contents are controlled to be 10 and 20 times higher than that of AlOOH-900 (Fig. S10), the dispersion threshold increases up to 9.9 wt.% and 19.1 wt.% on AlOOH-900-H_2_O_2_ and AlOOH-500 (Fig. 4c–e), respectively. The positive correlation between dispersion threshold and surface OH content (Fig. 4f) suggests that increasing OH content raises the dispersion capacity of γ-Al_2_O_3_ support by providing more anchoring sites for Cu species.

**Fig. 5:**
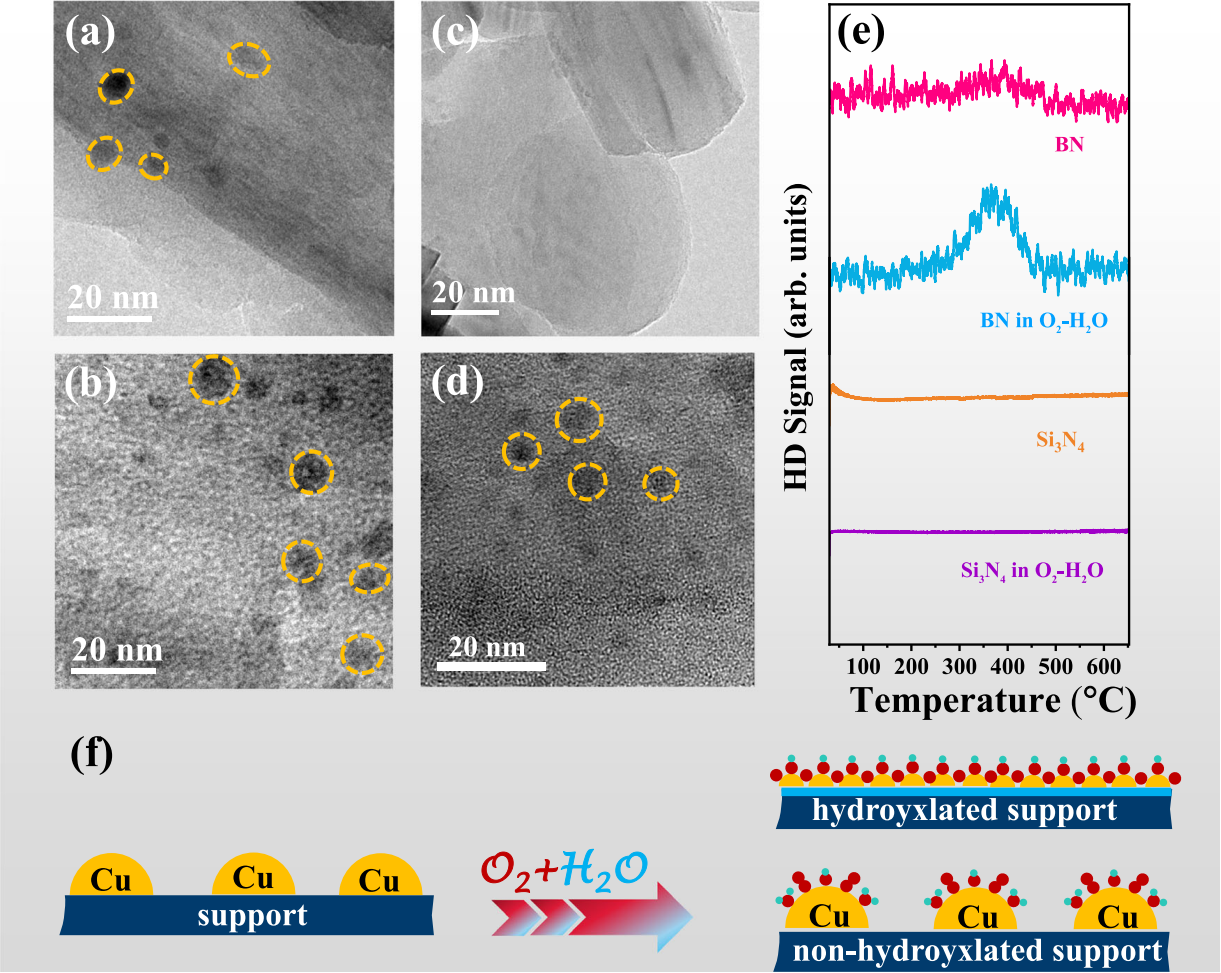
Support effect in the redispersion of Cu NPs at RT. TEM images of (**a**) 2Cu/BN, (**b**) 2Cu/Si_3_N_4_, (**c**) 2Cu/BN catalysts after O_2_-H_2_O treatment for 24 h, and (**d**) 2Cu/Si_3_N_4_ catalyst after O_2_-H_2_O treatment for 24 h. **e** H-D exchange curves of BN and Si_3_N_4_ supports after treatment in O_2_ and O_2_-H_2_O atmospheres for 24 h. **f** Scheme of support effect on water-assisted redispersion of Cu NPs at RT.

The role of surface OH on the redispersion of Cu NPs was further demonstrated by using various supports. TEM images in Fig. 5a, b show that Cu NPs with similar sizes around 6 nm exist on h-BN and Si_3_N_4_ surfaces. After O_2_-H_2_O treatment at RT for 24 h, no particles are observed on the surface of h-BN but a large number of Cu particles still remain on the surface of Si_3_N_4_ (Fig. 5c, d), which is also consistent with XRD data (Fig. S11). The results indicate that redispersion of Cu NPs can occur on h-BN surface but not on Si_3_N_4_ surface. Quasi in-situ XPS results show that Cu-OH species can form on the 2Cu/Si_3_N_4_ and 2Cu/h-BN samples after O_2_-H_2_O treatment (Fig. S12), and thus the different behavior is determined by the supports rather than mobile Cu-OH species. H-D exchange experiments on pure supports indicate that the O_2_-H_2_O treatment significantly increases the OH content on OH-free h-BN surface while no OH groups exist on Si_3_N_4_ surface even after the O_2_-H_2_O treatment (Fig. 5e). Subsequently, different oxide supports are selected to investigate the effect of surface hydroxylation on the redispersion of Cu NPs, including SiO_2_, ZrO_2_, TiO_2_, CeO_2_, and zeolites (MCM-41, SSZ-13 and H-beta), which can be hydroxylated (Fig. S13a–g), and nano BN which is not able to be hydroxylated (non-hydroxylated, Fig. S13h). XRD patterns show that Cu NPs can be redispersed on the hydroxylated supports (Fig. S14a–g) rather than the non-hydroxylated ones (Fig. S14h). The above results clearly confirm that surface hydroxylation is essential for the redispersion of Cu NPs.

Based on the above results we infer that the redispersion of Cu NPs under ambient conditions relies on two essential factors: the abundance of hydroxyl (OH) sites on the support surface and the generation of hydroxylated Cu-OH species. For supported Cu samples treated in O_2_, CuO_*x*_ species formed by oxidation in O_2_ may migrate on the γ-Al_2_O_3_ surface which however happen slowly at RT. In H_2_O-containing atmosphere, H_2_O significantly increases the hydroxylation degree of the γ-Al_2_O_3_ support, providing more anchoring sites and migration channels for surface Cu atoms. More crucially, the O_2_-H_2_O environment fosters the creation of more mobile hydroxylated Cu-OH species, which expedites the redispersion process (Fig. 3g). Thus, the role of H_2_O can be interpreted in two distinct manners: first, it promotes the formation of mobile hydroxylated Cu species (in the presence of O_2_) to speed up the migration of Cu species (kinetic aspect) (Fig. 3g); second, it enriches surface OH groups to provide more anchoring sites (thermodynamic aspect) (Fig. 5f). Consequently, the redispersion of Cu NPs under humid ambient conditions occurs through the synergistic promotion of H_2_O in both kinetics and thermodynamics.

### Effect of Cu redispersion on catalytic performance of RWGS and CO-PROX reactions

**Fig. 6:**
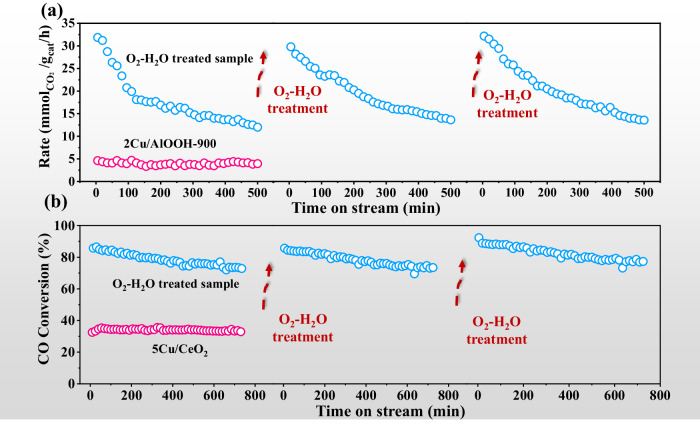
Catalytic performance of Cu-based catalysts. Stability test of (**a**) 2Cu/AlOOH-900 catalyst before and after O_2_-H_2_O treatment for RWGS reaction; **b** 5Cu/CeO_2_ catalyst before and after O_2_-H_2_O treatment for CO-PROX reaction. The deactivation-activation process can be repeated through O_2_-H_2_O treatment at RT. RWGS reaction condition: 450 °C, weight hourly space velocity (*WHSV*) = 36000 mL/g_cat_·h, 24%CO_2_/72%H_2_/4%N_2_, *P* = 0.1 MPa; CO-PROX reaction condition: 120 °C, *WHSV* = 36000 mL/g_cat_·h, 1% CO/0.5% O_2_/1% N_2_/97.5% H_2_, *P* = 0.1 MPa.

Due to its high CO selectivity and activity, as well as its low cost compared to gold and platinum, copper-based catalysts are one of the most promising candidates for RWGS reaction^49,50^. Fig. S15 shows that both fresh 2Cu/AlOOH-900 as well as the O_2_-H_2_O treated sample exhibit 100% selectivity towards CO at 450 °C. The fresh 2Cu/AlOOH-900 catalyst shows a CO_2_ conversion rate of $5 \, {{{\rm{mmol}}}}_{{{{\rm{CO}}}}_{2}}$/g_cat_/h and the CO_2_ conversion rate is greatly enhanced by up to 7 times on the O_2_-H_2_O treated sample (~ $34 \, {{{\rm{mmol}}}}_{{{{\rm{CO}}}}_{2}}$/g_cat_/h) (Fig. 6a), which is comparable to the highly dispersed copper catalysts in the literatures^49,50^. Since AlOOH-900 support before and after O_2_-H_2_O treatment shows no catalytic activity (Fig. S15), and thus the higher CO_2_ conversion rates obtained in the O_2_-H_2_O treated sample could be attributed to the better dispersion degree of Cu species. Unfortunately, the reaction rate decreases sharply from 34 to $10 \, {{\rm{mmol}}}_{{{{\rm{CO}}}}_{2}}$/g_cat_/h for 9 h at 450 °C and the reaction rate decreases in the second reaction cycle (Fig. S15). The obvious diffraction peaks of metallic Cu appear in the XRD spectra (Fig. S16 (i), (ii)), indicating that the highly dispersed Cu species have sintered during the reaction process. After treating the sintered Cu catalyst with O_2_-H_2_O at RT, the CO_2_ conversion rate returns to the initial state (~ $33 \, {{{\rm{mmol}}}}_{{{{\rm{CO}}}}_{2}}$/g_cat_/h). Interestingly, the deactivation-activation process can be repeated through cycles of high-temperature reaction and O_2_-H_2_O treatment at RT.

Copper-ceria is known as one of the most active catalysts for CO-PROX reaction at the temperature range of 100–140 °C, offering high CO conversion (60–100%) and O_2_ selectivity (60–100%)^51,52^. Figure 6b shows that O_2_-H_2_O treated sample exhibits higher reactivity compared to the fresh 5Cu/CeO_2_ (~ 35% CO conversion and ~ 47% O_2_ selectivity), achieving nearly 90% CO conversion and 72% O_2_ selectivity, demonstrating a competitive advantage compared to the published literatures^51,52^. Nevertheless, CO conversion of the O_2_-H_2_O treated sample drops from 89% to 72%, and the selectivity decreases from 74% to 56% after 12 h reaction, suggesting that the catalyst also undergoes Cu sintering in an H_2_-rich reaction atmosphere (confirmed by XRD in Fig. S16 ((iii), (iv))). Fortunately, the regeneration (both CO conversion and O_2_ selectivity) can be easily achieved through simple O_2_-H_2_O treatment at RT (Fig. 6b, Fig. S17). The reaction results that the Cu-based catalysts undergo sintering and deactivation during the reaction processes, while O_2_-H_2_O treatment at RT can serve as an effective reactivation method to regenerate the catalysts.

The redispersion of Cu NPs into highly dispersed Cu^2+^ species in O_2_-H_2_O contributes to the enhanced performance and recovered activity for RWGS and CO-PROX reactions. Besides, the redispersion of Cu NPs provides an effective method to tune the size of Cu catalyst, which can be applied in various scenarios, such as Cu NPs for CO oxidation^53^ and isolated Cu atoms for selective catalytic reduction of NO_*x*_ with NH_3_ (NH_3_-SCR) reaction^54^ (Fig. S18).

## Discussion

Spontaneous redispersion of aggregated Cu particles can occur on γ-Al_2_O_3_ surface in the humid environment at RT. The formation of hydroxylated Cu species and enriched surface OH groups under the condition are the two key factors for the facile redispersion of Cu NPs. Hydroxylated Cu species, generated in O_2_-H_2_O atmosphere, act as a mobile species, enabling the copper diffusion to be kinetically feasible at RT. On the other hand, the highly hydroxylated surface induced by H_2_O provides numerous anchoring sites of OH for diffusing Cu species, facilitating an energy-favorable configuration of highly dispersed Cu species. The treated Cu catalysts exhibit better performance in the RWGS and CO-PROX reactions. Most notably, sintering of the Cu-based catalysts during reactions can be easily reversed through simple O_2_-H_2_O treatment at RT. This work provides an effective method for the regeneration of sintered Cu catalysts as well as deepens the understanding of the role of both atmosphere and support in the redispersion of metal particles under relatively mild conditions.

## Methods

### Sample preparation

γ-Al_2_O_3_ supports were prepared by calcination of pseudo-boehmite at 500 and/or 900 °C, at a heating rate of 2 °C·min^–1^ and a dwell time of 5 h (denoted as AlOOH-*T*, in which T represents the calcination temperature)^14^. To increase the surface OH sites, the AlOOH-*T* sample was subject to treatment in hydrogen peroxide (H_2_O_2_) solution at RT for 24 h (AlOOH-*T*-H_2_O_2_)^14^. Commercially available supports, such as h-BN (98.5% purity, 1 μm, Shanghai Aladdin Biochemistry technology Co., Ltd.), Nano BN (99.9% purity, <200 nm, Shanghai Macklin biochemical Co., Ltd.), ZrO_2_ (99.9% purity, ≤ 100 nm, Shanghai Aladdin Biochemistry technology Co., Ltd.), TiO_2_ (99.8% purity, rutile, 40 nm, Shanghai Aladdin Biochemistry technology Co., Ltd.), Si_3_N_4_ (99.3% purity, 325 mesh, Alfa Aesar, USA.), CeO_2_ (99.9% purity, 50 nm, Shanghai Macklin biochemical Co., Ltd.), SSZ-13 (3–10 μm, Dalian Ze’er Catalytic Materials Co., Ltd.), H-beta (2–5 μm powder, Si/Al = 25–30, Dalian Ze’er Catalytic Materials Co., Ltd.), MCM-41 (3–6 μm powder, Pure Silica, Dalian Ze’er Catalytic Materials Co., Ltd.), were used directly. Copper hydroxide (Cu(OH)_2_) was purchased from Macklin (99.9% purity, ~ 40 nm) and directly used for the synthesis. 2Cu/AlOOH-900 catalyst was synthesized via conventional wet impregnation. Briefly, AlOOH-900 (2 g) was impregnated with an aqueous solution of Cu(NO_3_)_2_·3H_2_O (1.56 mL, 0.4 mol/L), corresponding to a copper weight loading of 2 wt.%. The resulting sample was dried overnight and calcined at 500 °C for 4 h in air, followed by reduction in pure H_2_ at 500 °C for 2 h with a flow rate of 100 mL/min. Cu-based catalysts with different Cu weight loadings supported on different supports were prepared according to the above-mentioned methods, and an equal proportion of Cu(NO_3_)_2_·3H_2_O solution was added to 2 g of the supports (for example, 10Cu/AlOOH-900 need to add 7.81 mL Cu(NO_3_)_2_·3H_2_O solution). After reduction in H_2_ at 500 °C for 2 h, the obtained sample was denoted as *x*Cu/support, where *x* represents the weight loading of Cu. The reduced samples were further treated in different gases such as O_2_, Ar, and H_2_O with a flow rate of 100 mL/min at RT for 24 h. AlOOH-900 (2 g) and Cu(OH)_2_ powders (15 mg, Cu weight loading 0.5 wt.%) were physically mixed, and the obtained Cu(OH)_2_-AlOOH-900 sample was labeled as PM (Physical mixture). Cu(OH)_2_-AlOOH-900 sample was then vigorously stirred in 50 ml water for 24 h at RT before being evaporated at 100 °C to remove the water, and the obtained sample was labeled as IM (Impregnation sample). Water vapor was introduced by passing the gas flow through a bubbler with about 3 vol.% H_2_O at RT.

### Sample Characterizations

X-ray diffraction (XRD) patterns were acquired using an Empyrean-100 diffractometer equipped with a Cu Kα radiation source (λ = 1.5418 Å) at 40 kV and 40 mA. Quasi in-situ electron paramagnetic resonance (EPR) spectra were collected at 110 K using a Bruker A200 EPR spectrometer. Ultraviolet-visible (UV-Vis) spectra were acquired in Lambda 950 (Perkin Elmer) equipped with an in-situ reaction cell, in normal or time-dependent modes. Catalyst powders (30–50 mg) were pressed into self-supporting wafers and placed within a temperature-controlled stainless-steel cell equipped with CaF_2_ windows and connected to a gas manifold. The sample was pretreated at 500 °C for 2 h under a H_2_ atmosphere with a flow rate of 100 mL/min to obtain the first spectrum, and then the corresponding gas was introduced for 30 min before collection of the next spectrum. High-resolution transmission electron microscopy (HR-TEM) images were recorded on a JEOL JEM 2100 TEM instrument with a 200 kV accelerating voltage. High-angle annular dark-field scanning transmission electron microscopy (HAADF-STEM) images were acquired using a field emission TEM (JEM-F200) with a 200 kV operating voltage. HAADF-STEM images were obtained on a JEM ARM 300 F with a 300 kV accelerating voltage.

*Q*uasi in-situ X-ray photoelectron spectroscopy (XPS) measurements were carried out with a spectrometer equipped with an Mg Kα X-ray source operated at 300 W. Samples were treated in a reaction chamber at ambient pressure and then transferred to the analysis chamber immediately without exposure to air. All XPS binding energy peaks were calibrated by C 1 *s* at 284.6 eV. *Q*uasi in-situ X-ray absorption spectroscopy (XAS) spectra were collected in fluorescence mode at RT at the BL11B beamline of Shanghai Synchrotron Radiation Facility (SSRF). Before measurement, the sample treated for different times was sealed in capillary tubes in the glove box without exposure to air.

H-D exchange experiments were conducted in a homemade microreactor connected with a mass spectrometry (OMNI Star TM). Typically, 0.1 g sample was loaded in a quartz tube and then pretreated under Ar atmosphere at 200 °C for 2 h. After the pretreatment, the H-D exchange experiment was started with a heating rate of 10°/min from RT to 750 °C by recording the mass spectroscopy HD signal (*m*/*z* = 3).

### Catalytic Test

Reverse water gas shift (RWGS) reactions were tested using a homemade fixed-bed micro-reactor with the weight hourly space velocity (*WHSV*) of 36,000 mL/g_cat_·h. The 50 mg pelleting catalysts (20 ~ 40 mesh) were loaded in a quartz tube with an inner diameter of 6 mm. The reaction gas contains 24% CO_2_, 72% H_2_ (volume ratio), balanced with N_2_, which was used as an internal standard. Before measurement, the fresh 2Cu/AlOOH-900 catalyst was pretreated by H_2_ at 500 °C for 1 h to reduce the oxidized Cu species on the sample surface. The O_2_-H_2_O treated sample was directly used for RWGS reaction test. The products were analyzed by an online gas chromatograph (Agilent 490 Micro GC) equipped with a 5 Å molecular sieve column and a thermal conductivity detector. The reaction took place at atmospheric pressure and 450 °C. Only CO is generated during the reaction, and CO_2_ conversion and CO selectivity were calculated according to the following equations:

1 $${{{\mbox{CO}}}}_{2}\,{{\mbox{Conversion}}}(\%)=\frac{{{{\mbox{C}}}}_{{{{\mbox{CO}}}}_{2}({{\mbox{inlet}}})}-{{{\mbox{C}}}}_{{{{\mbox{CO}}}}_{2}({{\mbox{outlet}}})}}{{{{\mbox{C}}}}_{{{{\mbox{CO}}}}_{2}({{\mbox{inlet}}})}}\times 100\%$$

2 $${{\mbox{CO}}}\,{{\mbox{Selectivity}}}(\%)=\frac{{{{\mbox{C}}}}_{{{\mbox{CO}}}({{\mbox{outlet}}})}}{{{{\mbox{C}}}}_{{{\mbox{CO}}}({{\mbox{outlet}}})}+{{{\mbox{C}}}}_{{{{\mbox{CH}}}}_{4}({{\mbox{outlet}}})}}\times 100\%$$

where the subscripts “inlet” and “outlet” are related to the inlet and outlet gas concentrations, respectively.

Preferential oxidation of CO in excess of H_2_ (CO-PROX) reactivity was tested in a fixed-bed reactor by using 50 mg of sieved catalyst (20–40 mesh) in a gas mixture of 1% CO/0.5% O_2_/1% N_2_/97.5% H_2_ (volume ratio) with a *WHSV* of 36, 000 mL/g_cat_·h. Prior to the measurement, the fresh sample was pretreated in pure H_2_ at 500 °C for 1 h. The reaction was performed at 120 °C and the products were analyzed by an online gas chromatograph (Agilent 490 Micro GC) equipped with a 5 Å molecular sieve column and a thermal conductivity detector. The CO conversion and O_2_ selectivity were calculated according to the following equations:

3 $${{\mbox{CO}}}\,{{\mbox{Conversion}}}(\%)=\frac{{{{\mbox{C}}}}_{{{\mbox{CO}}}({{\mbox{inlet}}})}-{{{\mbox{C}}}}_{{{\mbox{CO}}}({{\mbox{outlet}}})}}{{{{\mbox{C}}}}_{{{\mbox{CO}}}({{\mbox{inlet}}})}}\times 100\%$$

4 $${{{\mbox{O}}}}_{2}\,{{\mbox{Selectivity}}}(\%)=\frac{0.5\times ({{{\mbox{C}}}}_{{{\mbox{CO}}}({{\mbox{inlet}}})}-{{{\mbox{C}}}}_{{{\mbox{CO}}}({{\mbox{outlet}}})})}{({{{\mbox{C}}}}_{{{{\mbox{O}}}}_{2}({{\mbox{inlet}}})}-{{{\mbox{C}}}}_{{{{\mbox{O}}}}_{2}({{\mbox{outlet}}})})}\times 100\%$$

where the subscripts “inlet” and “outlet” are related to the inlet and outlet gas concentrations, respectively.

CO oxidation reaction was tested in a fixed-bed reactor at atmospheric pressure with *WHSV* of 40,000 mL/g_cat_·h. The reaction gas contains 1% CO, 20% O_2_, 1% N_2_, and 78% He. The products were analyzed by an online gas chromatograph (Agilent 490 Micro GC) equipped with a 5 Å molecular sieve column and a thermal conductivity detector.

5 $${{\mbox{CO}}}\,{{\mbox{Conversion}}}(\%)=\frac{{{{\mbox{C}}}}_{{{\mbox{CO}}}({{\mbox{inlet}}})}-{{{\mbox{C}}}}_{{{\mbox{CO}}}({{\mbox{outlet}}})}}{{{{\mbox{C}}}}_{{{\mbox{CO}}}({{\mbox{inlet}}})}}\times 100\%$$

where the subscripts “inlet” and “outlet” are related to the inlet and outlet gas concentrations, respectively.

Selective catalytic reduction of NO_*x*_ with NH_3_ (NH_3_-SCR) activity tests of the sieved powder catalysts were carried out in a fixed-bed quartz flow reactor at atmospheric pressure with *WHSV* of 120,000 mL/g_cat_·h. The reaction gas contains 500 ppm NO, 500 ppm NH_3_, 4 % O_2_, and balance N_2_. The effluent gases including NO, NH_3_, NO_2_, and N_2_O were continuously analyzed by an online Nicolet iS50 IR spectrometer (Nicolet, USA) equipped with a gas cell. The activity data were recorded at every target temperature after stabilizing for 60 min. Finally, NO_*x*_ conversion was calculated using the below equations.

6 $${{{\mbox{NO}}}}_{x}\,{{\mbox{Conversion}}}(\%)=\frac{{{{\mbox{C}}}}_{{{{\mbox{NO}}}}_{x}({{\mbox{inlet}}})}-{{{\mbox{C}}}}_{{{{\mbox{NO}}}}_{x}({{\mbox{outlet}}})}}{{{{\mbox{C}}}}_{{{{\mbox{NO}}}}_{x}({{\mbox{inlet}}})}}\times 100\%$$

where the subscripts “in” and “out” are related to the inlet and outlet gas concentrations, respectively.

### Supplementary information

Supplementary Information

Peer Review File

### Source data

Source Data

## Data Availability

All data that support the findings in this paper are available within the article and its Supporting Information or are available from the corresponding authors upon reasonable request. Source data are provided with this paper.
