# Supplementary material for: Water-assisted oxidative redispersion of Cu particles through formation of Cu hydroxide at room temperature
Source: Nat Commun. 2024 Apr 8;15:3046. doi: 10.1038/s41467-024-47397-z (PMC11001857; doi:10.1038/s41467-024-47397-z)
Supplement: Supplementary file 1 — Supplementary Information [file 41467_2024_47397_MOESM1_ESM.pdf]

Supporting Information

Water-assisted room temperature oxidative redispersion of Cu particles through formation of Cu hydroxide

Yamei Fan[1,2,5], Rongtan Li[2,5], Beibei Wang[3], Xiaohui Feng[1,2], Xiangze Du[2], Chengxiang Liu[2], Fei Wang[4], Conghui Liu[2], Cui Dong[2], Yanxiao Ning[2], Rentao Mu[2], Qiang Fu[2*]

[1]Department of Chemical Physics, University of Science and Technology of China, Hefei, 230026, China

[2]State Key Laboratory of Catalysis, Chinese Academy of Sciences, Dalian Institute of Chemical Physics, Dalian, 116023, China

[3]Center for Transformative Science, ShanghaiTech University, Shanghai, 201210, China

[4]Faculty of Environmental Science and Engineering, Kunming University of Science and Technology, Kunming, 650500, China

[5]These authors contributed equally: Yamei Fan, Rongtan Li

*Email: qfu@dicp.ac.cn

Table of Contents

Supplementary Fig. 1 XRD patterns of γ-Al_2O_3 supports. 3

Supplementary Fig. 2 XRD patterns of 2Cu/AlOOH-900. 4

Supplementary Fig. 3 Extended X-ray absorption fine structure (EXAFS) analysis. ... 5

Supplementary Fig. 4 X-ray absorption near edge structure (XANES) analysis. 6

Supplementary Fig. 5 OH content of γ-Al_2O_3 samples treated in O_2-H_2O and Ar-H_2O atmospheres. 8

Supplementary Fig. 6 Cu L_3VV Auger spectra. 9

Supplementary Fig. 7 Quasi *in-situ* Cu 2*p* XPS spectra. 10

Supplementary Fig. 8 EPR spectra. 11

Supplementary Fig. 9 XRD spectra of $Cu(OH)_2$-AlOOH-900... 12

Supplementary Fig. 10 OH content of different γ-Al_2O_3 samples. 12

Supplementary Fig. 11 XRD patterns of 2Cu/BN and 2Cu/Si_3N_4. 14

Supplementary Fig. 12 Cu $2p_{3/2}$ XPS spectra of 2Cu/BN and 2Cu/Si_3N_4.. 15

Supplementary Fig. 13 H-D exchange profiles of other supports. 15

Supplementary Fig. 14 XRD patterns of Cu/supports. 17

Supplementary Fig. 15 RWGS reaction performance. 17

Supplementary Fig. 16 XRD patterns of Cu-based catalysts. 18

Supplementary Fig. 17 O_2 selectivity of CO-PROX reaction. 19

Supplementary Fig. 18 Catalytic activity of CO oxidation and NH_3-SCR reaction. 20

Table S1. EXAFS distance and fitting parameters for the Cu-based catalysts. 21

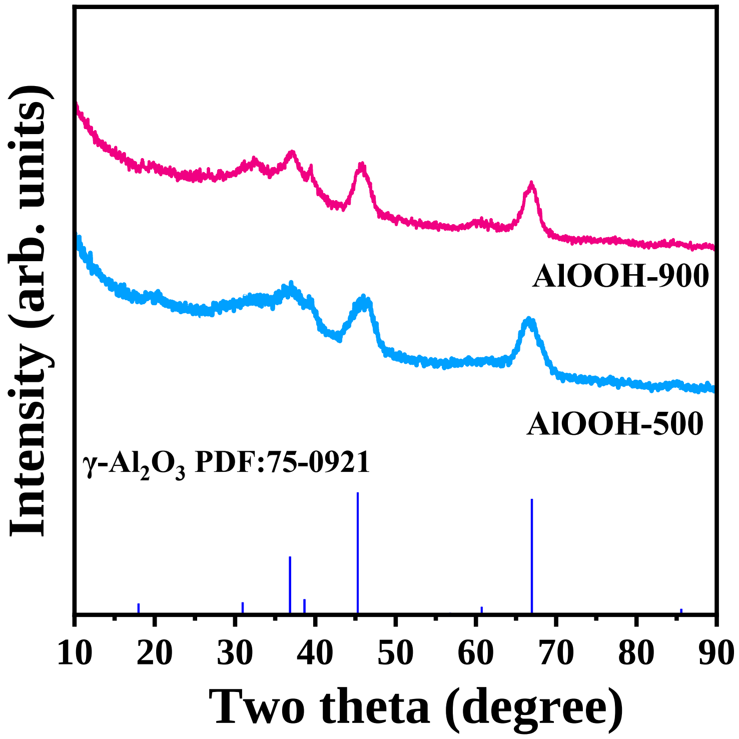

Supplementary Fig. 1 XRD patterns of γ-Al_2O_3 supports. XRD spectra of AlOOH-*T* (*T* = 500/900).

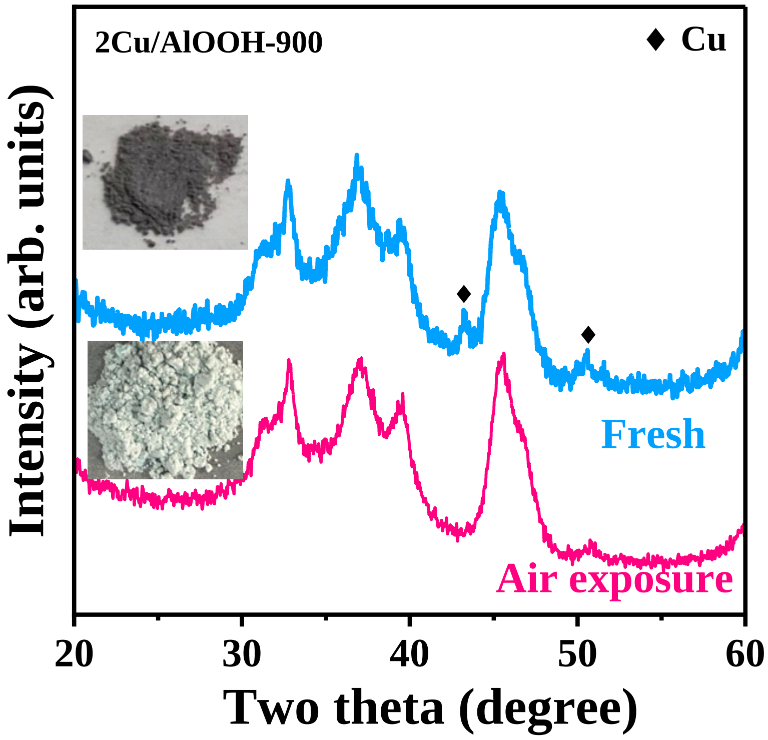

Supplementary Fig. 2 XRD patterns of 2Cu/AlOOH-900. The sky-blue line denotes the fresh 2Cu/AlOOH-900 sample, while the rose-colored line signifies the 2Cu/AlOOH-900 sample after exposure to air.

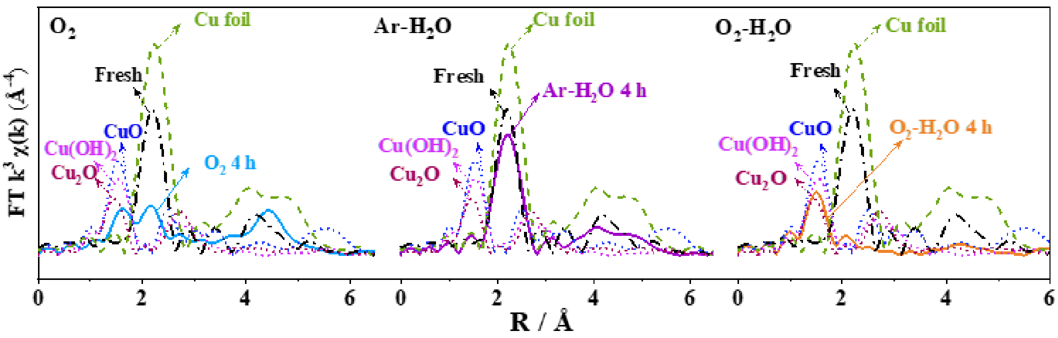

Supplementary Fig. 3 Extended X-ray absorption fine structure (EXAFS) analysis. Spectra of 2Cu/AlOOH-900 treated in O_2, Ar-H_2O, O_2-H_2O for 4 h, and standard samples of Cu foil, Cu_2O, CuO and $Cu(OH)_2$.

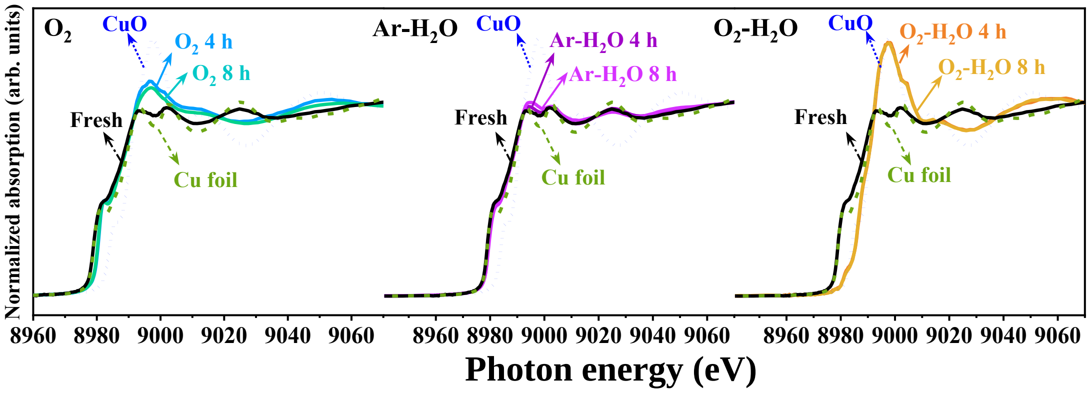

Supplementary Fig. 4 X-ray absorption near edge structure (XANES) analysis. Cu K-edge XANES spectra of Cu NPs treated in O_2, Ar-H_2O, O_2-H_2O for 4 h, 8 h, and standard samples of Cu foil and CuO.

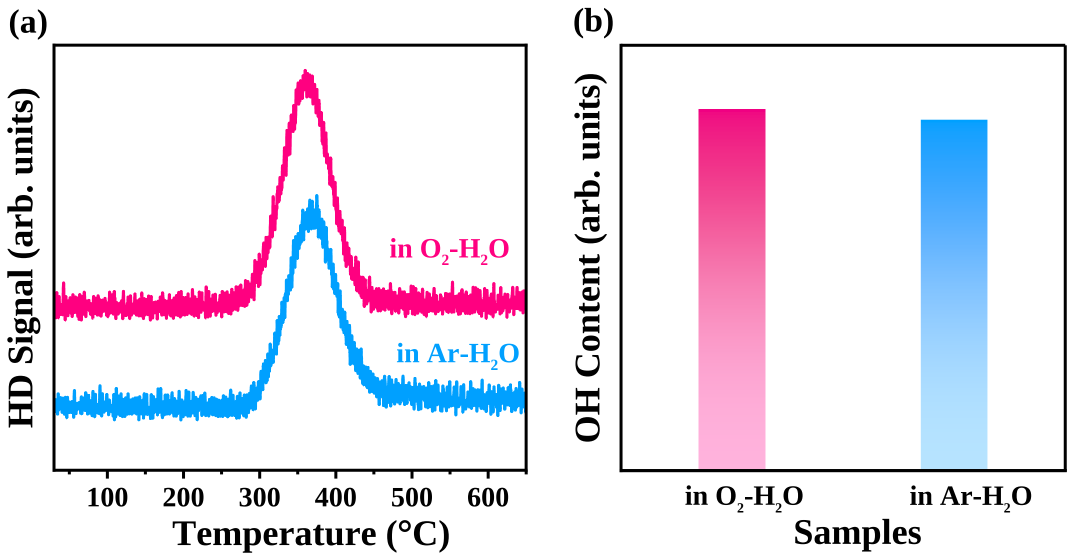

Supplementary Fig. 5 OH content of γ-Al_2O_3 samples treated in O_2-H_2O and Ar-H_2O atmospheres. (a) H-D exchange profiles of AlOOH-900 treated in O_2-H_2O and Ar-H_2O atmospheres. (b) OH content of AlOOH-900 samples determined from the peak area of H-D curves normalized by support mass.

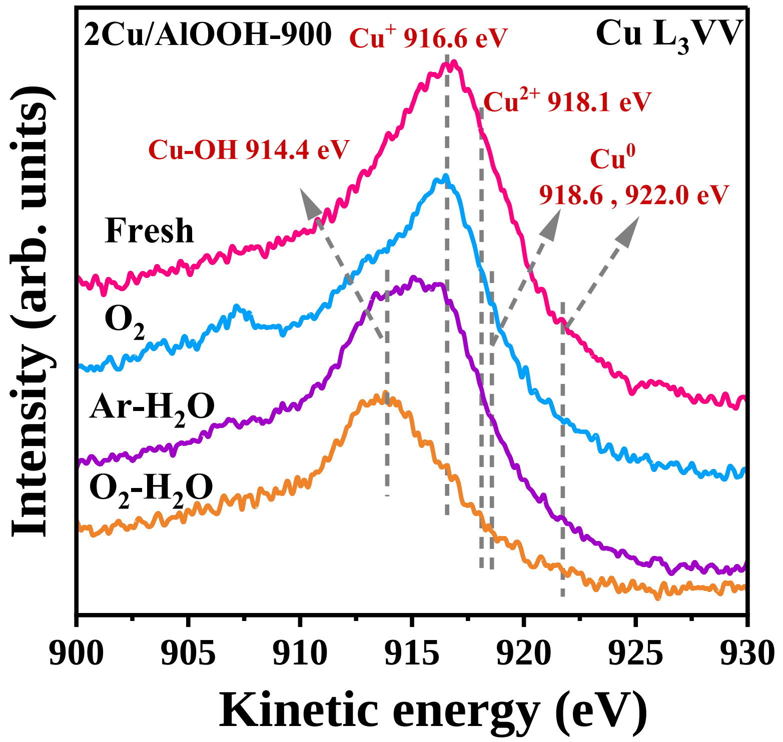

Supplementary Fig. 6 Cu L3VV Auger spectra. 2Cu-AlOOH-900 and samples treated in O_2, Ar-H_2O and O_2-H_2O atmospheres at RT for 4 h.

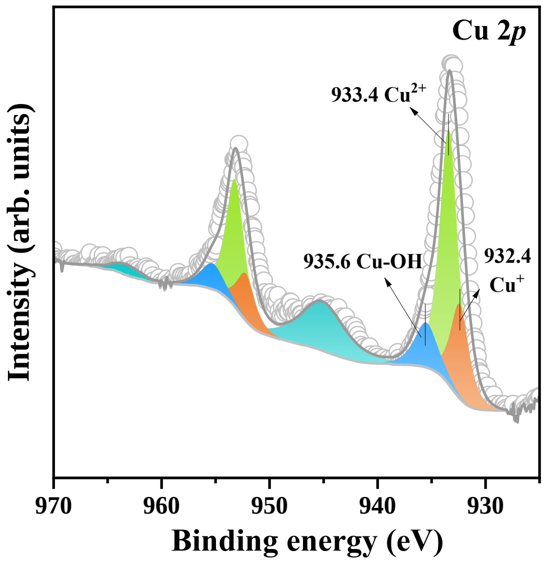

Supplementary Fig. 7 Quasi *in-situ* Cu 2*p* XPS spectra. Cu 2*p* XPS spectra of 2Cu/AlOOH-900 treated in O_2 for 24 h and then treated in Ar-H_2O for 24 h.

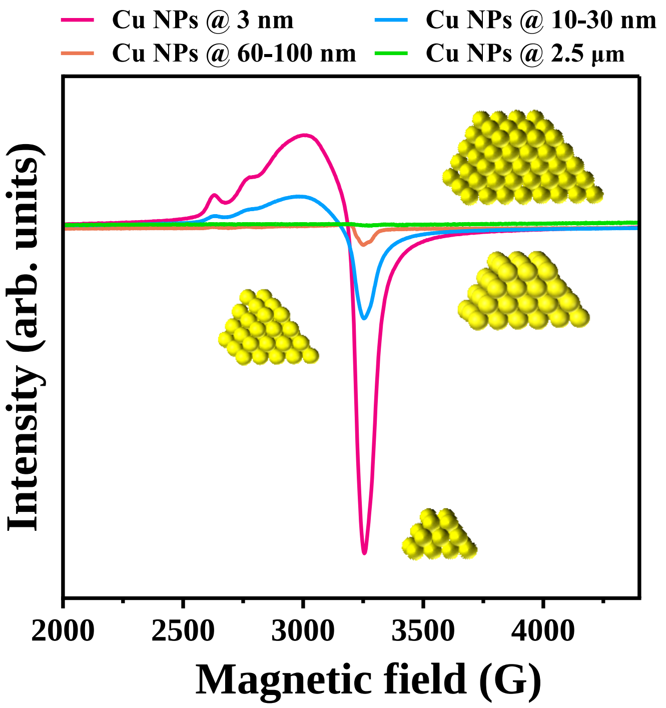

Supplementary Fig. 8 EPR spectra. Cu particles with different sizes supported on AlOOH-900 after O_2-H_2O treatment at RT for 24 h.

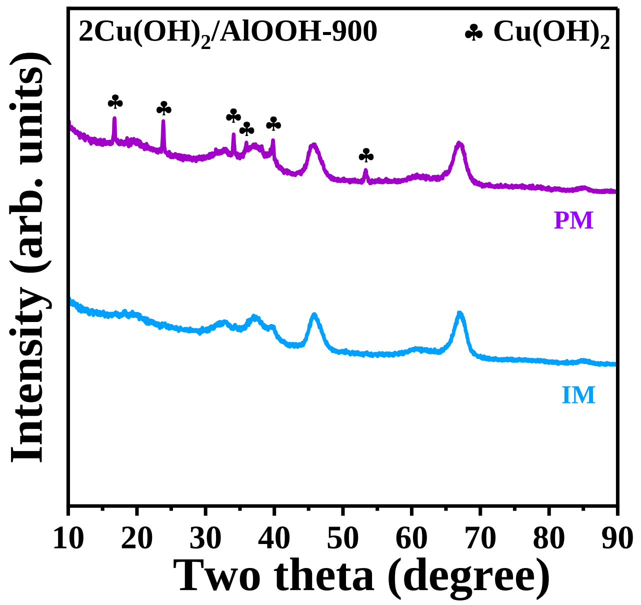

Supplementary Fig. 9 XRD spectra of $Cu(OH)_2$-AlOOH-900. The purple line denotes the fresh $Cu(OH)_2$-AlOOH-900 sample, while the sky blue line signifies the $Cu(OH)_2$-AlOOH-900 sample after water impregnation.

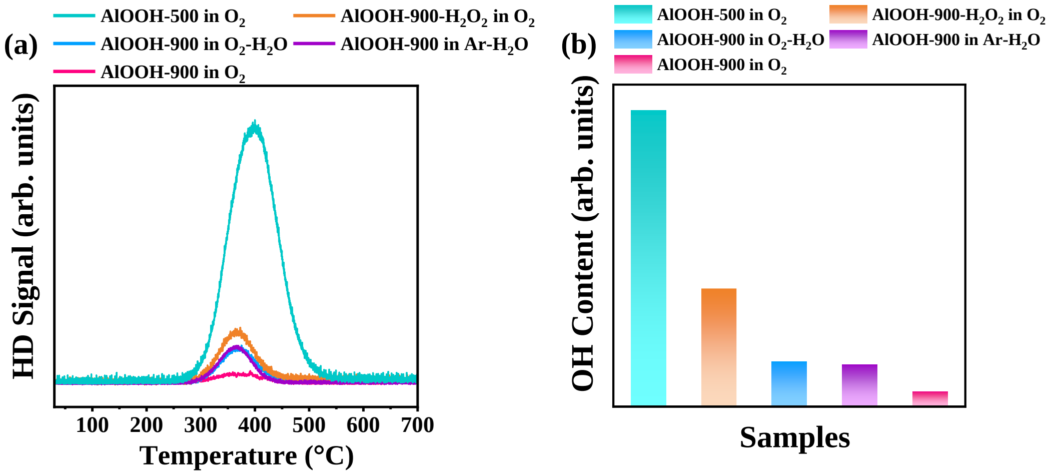

Supplementary Fig. 10 OH content of different γ-Al_2O_3 samples. (a) H-D exchange profiles of AlOOH-900 samples treated in different processes. (b) OH contents of different AlOOH-900 samples determined from the peak area of H-D curves normalized by support mass.

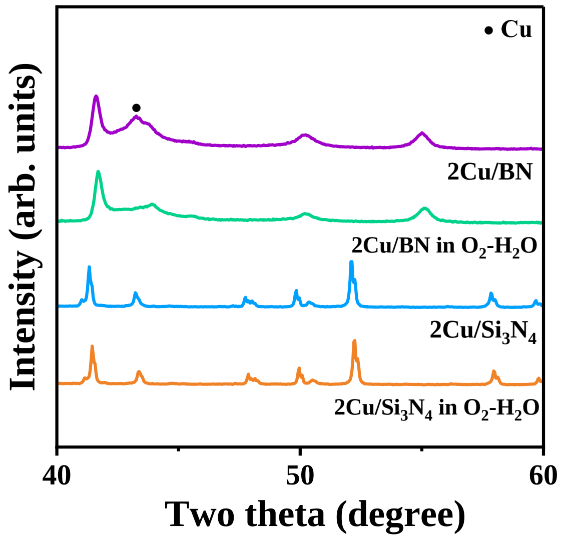

Supplementary Fig. 11 XRD patterns of 2Cu/BN and 2Cu/Si_3N_4. 2Cu/BN and 2Cu/Si_3N_4 before and after treatment in O_2-H_2O at RT for 24 h.

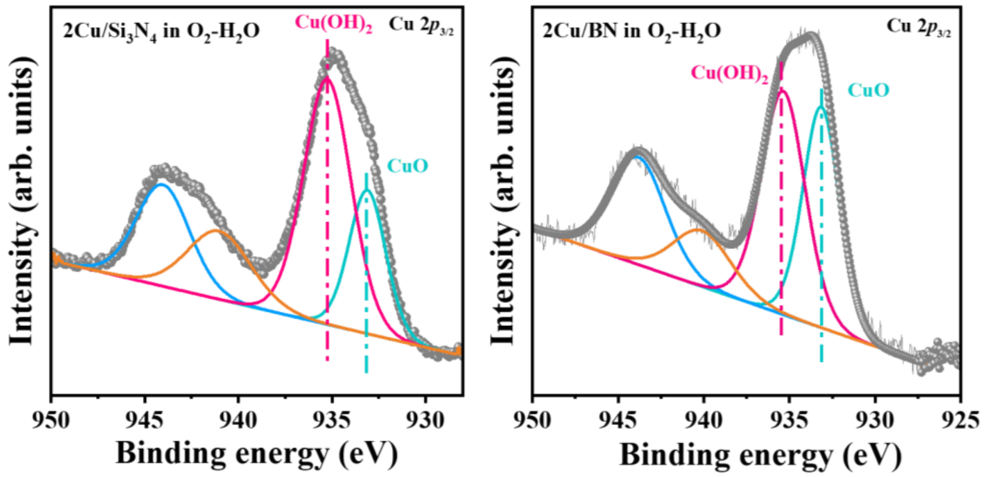

Supplementary Fig. 12 Cu 2$p_{3/2}$ XPS spectra of 2Cu/Si_3N_4 and 2Cu/BN. (a) 2Cu/Si_3N_4, (b) 2Cu/BN after treatment in O_2-H_2O at RT for 24 h.

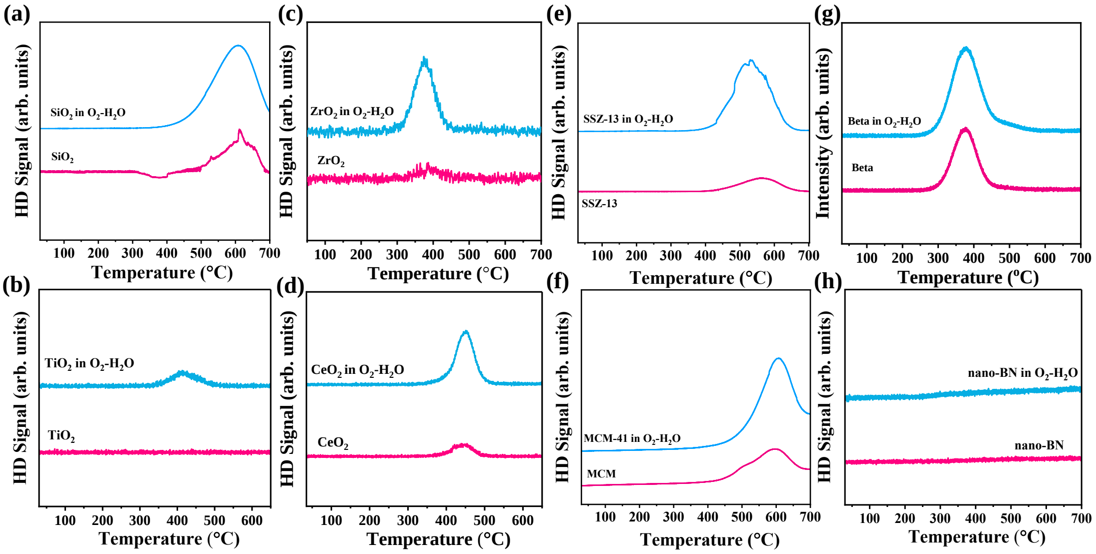

Supplementary Fig. 13 H-D exchange profiles of supports. (a) SiO_2, (b) TiO_2, (c) ZrO_2, (d) CeO_2, (e) SSZ-13, (f) MCM-41, (g) H-Beta and (h) nano BN before and after exposure to O_2-H_2O at RT for 24 h.

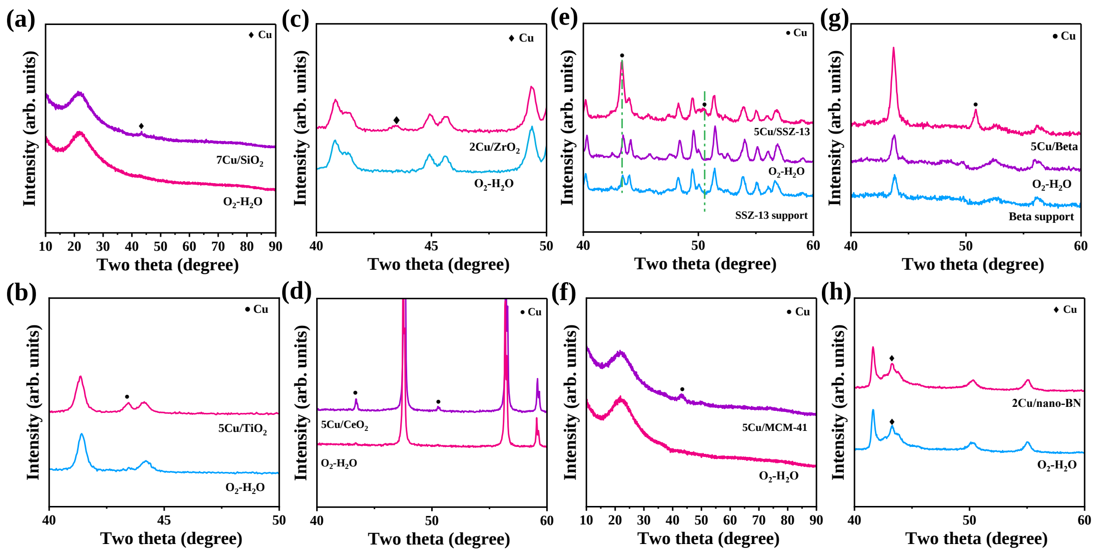

Supplementary Fig. 14 XRD patterns of Cu/supports. (a) 7Cu/SiO_2, (b) 5Cu/TiO_2, (c) 2Cu/ZrO_2, (d) 5Cu/CeO_2, (e) 5Cu/SSZ-13, (f) 5Cu/MCM-41, (g) 5Cu/H-Beta and (h) 2Cu/nano BN before and after exposure to O_2-H_2O at RT for 24 h.

The different Cu loadings on different supports are determined by the dispersion threshold of different supports under experimental conditions.

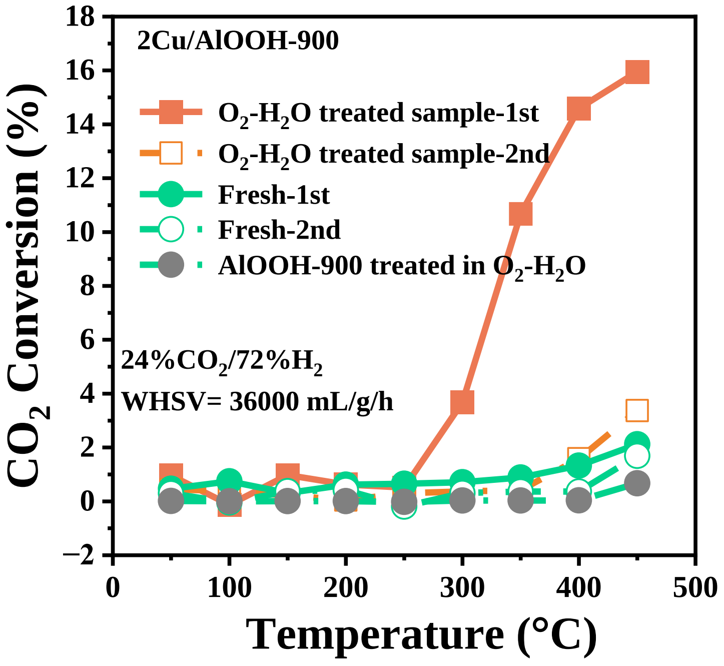

Supplementary Fig. 15 RWGS reaction performance. 2Cu/AlOOH-900 before and after exposure to O_2-H_2O. Reaction condition: weight hourly space velocity (WHSV) = 36000 mL/g_{cat}·h, 24%CO_2/72%H_2/4%N_2, P = 0.1 MPa.

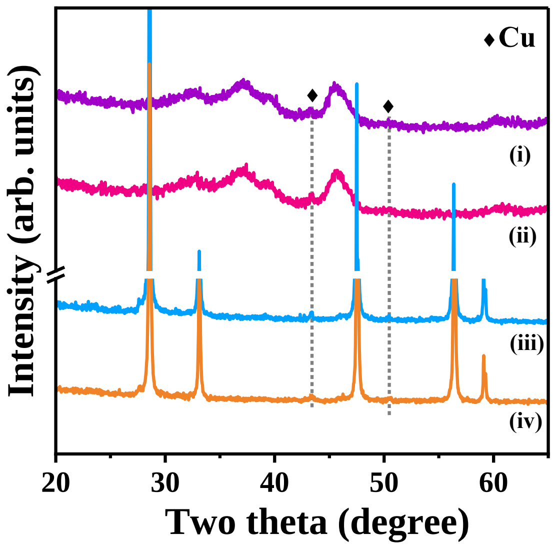

Supplementary Fig. 16 XRD patterns of Cu-based catalysts. (i) 2Cu/AlOOH-900, (ii) 2Cu/AlOOH-900 treated in O_2-H_2O after RWGS reaction as well as (iii) 5Cu/CeO_2, (iv) 5Cu/CeO_2 treated in O_2-H_2O after CO-PROX reaction.

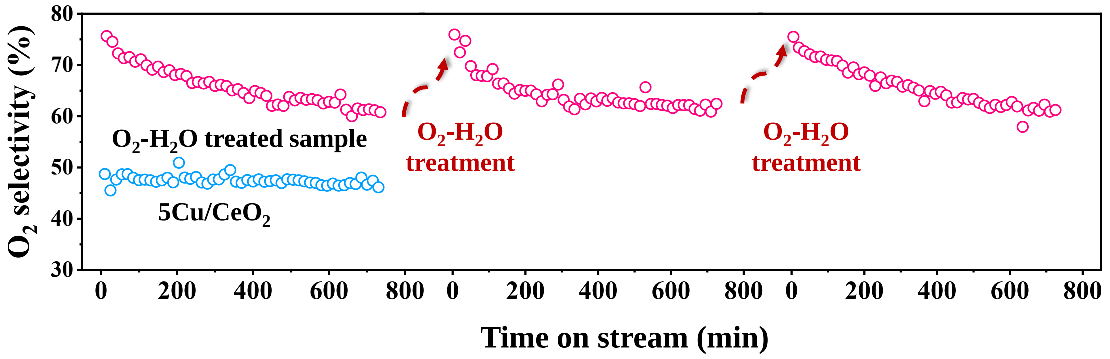

Supplementary Fig. 17 O_2 selectivity of CO-PROX reaction. Time-on-stream experiments of O_2 selectivity of CO-PROX reaction over $5Cu/CeO_2$ catalysts before and after O_2-H_2O treatment at RT for 24 h. The deactivation-activation process can be cyclically repeated through O_2-H_2O treatment at RT. CO-PROX reaction condition: 120 °C, WHSV = 36, 000 mL/g_{cat}·h, 1% CO/0.5% O_2/1% N_2/97.5% H_2, P = 0.1 MPa.

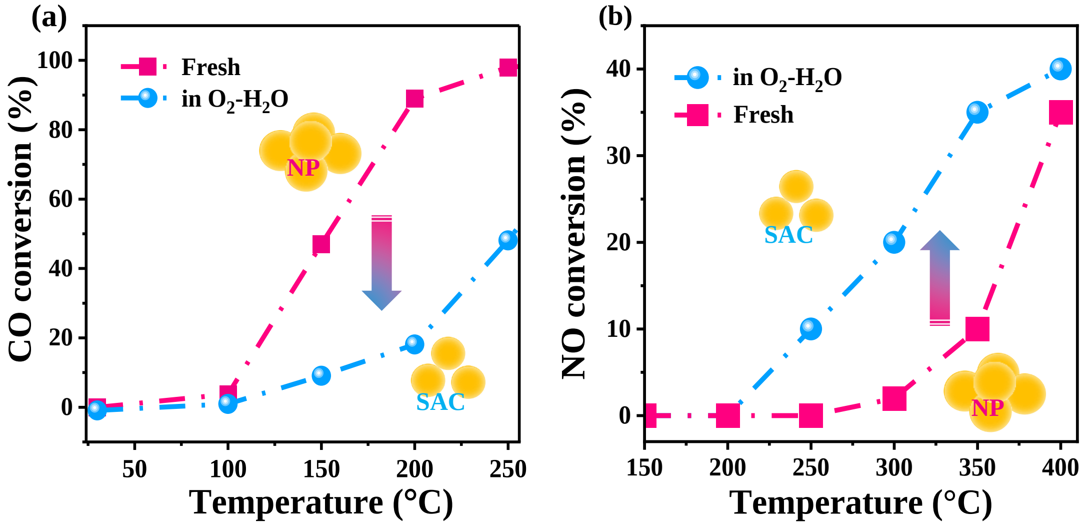

Supplementary Fig. 18 Catalytic activity of CO oxidation and NH_3-SCR reaction. (a) CO oxidation activity over 2Cu/AlOOH-900 catalysts before and after O_2-H_2O treatment at RT for 24 h. (b) NO_x conversion for NH_3-SCR reaction over 2Cu/AlOOH-900 catalysts before and after O_2-H_2O treatment at RT for 24 h. CO condition: WHSV = 40, 000 mL/g_{cat}·h, 1% CO/20 %O_2/4% N_2, P = 0.1 MPa; Selective catalytic reduction of NO_x with NH_3 (NH_3-SCR) reaction condition: WHSV = 120, 000 mL/g_{cat}·h, 500 ppm NO, 500 ppm NH_3, 4 % O_2, and balance N_2, P = 0.1 MPa.

Table S1. EXAFS distance and fitting parameters for the Cu-based catalysts.

Fitting parameters: S_0^2 = 0.88 calculated using a Cu foil standard; k range: 2.3-11 $Å^{-1}$.

Sample	Shell	R (Å)[a]	CN[b]	$\triangle E_0$ (eV)[c]	σ^2 (10^{-3} $Å^2$)[d]	R factor (%)
O_2-H_2O-4 h	Cu-O	1.94 (0.02)	3.0 (0.4)	2.1 (2.2)	3.7	1.1
O_2-4 h	Cu-O	1.96 (0.03)	2.0 (0.4)	9.8 (1.9)	5.4	1.8
	Cu-Cu	2.34 (0.05)	1.4 (0.5)	8.6 (2.3)	5.2	
Ar-H_2O-4 h	Cu-Cu	2.53 (0.08)	6.6 (0.6)	2.4 (1.4)	8.3	1.0
2Cu/AlOOH-900	Cu-Cu	2.51 (0.05)	7.0 (0.8)	0.5 (0.8)	8.2	1.7

[a]Radical distance; [b]Coordination number; [c]Energy correction; [d]Debye–Waller factor
